# Long-Term Effects of an Oligoantigenic Diet in Children with Attention-Deficit/Hyperactivity Disorder (ADHD) on Core Symptomatology

**DOI:** 10.3390/nu14235111

**Published:** 2022-12-01

**Authors:** Greta Walz, Nicola Blazynski, Lukas Frey, Katja Schneider-Momm, Hans-Willi Clement, Reinhold Rauh, Eberhard Schulz, Monica Biscaldi, Christina Clement, Christian Fleischhaker

**Affiliations:** Department of Child and Adolescent Psychiatry, Psychotherapy and Psychosomatics, Medical Center—University of Freiburg, Faculty of Medicine, University of Freiburg, D-79104 Freiburg, Germany

**Keywords:** attention-deficit/hyperactivity disorder, child, adolescents, oligoantigenic diet, nutrition, food intolerance, diet, behavior, elimination, follow-up

## Abstract

In the early 1920s, it was discovered that nutrition is associated with what is known today as Attention-Deficit/Hyperactivity Disorder (ADHD) and that certain foods can worsen the symptoms. In previous studies, approximately 60% of the participants experience at least a 40% reduction in ADHD symptoms after an oligoantigenic diet (OD). The purpose of this study was to evaluate ADHD symptoms in children approximately 3.5 years after completing a 4-week oligoantigenic diet. Among 28 participants who completed the 4-week diet, 21 were re-assessed for this study after 3.5 years. The severity of ADHD symptoms was assessed with the ADHD-Rating-Scale-IV (ARS). Of 21 participants, 14 fulfilled the responder criterion, whereas 7 did not. At follow-up, 28% of the participants were taking medication. The mean ARS total score improved significantly from T1: *M* = 29.62 (*SD* = 9.80) to T2: *M* = 15.86 (*SD* = 8.56) between the time points before and after the diet (*d* = −1.91). There was also a lower ARS total score at the follow-up T5: *M* = 16.00 (*SD* = 10.52) compared to before the diet (*d* = −1.17). This study shows that individually adjusted nutrition significantly improved the ADHD symptomatology of the participants long-term. This suggests that an oligoantigenic diet with subsequent individual nutritional recommendations could become an additional treatment option for children with ADHD.

## 1. Introduction

Worldwide, 5–10% of all children and 4% of all adults are affected by attention-deficit/hyperactivity disorder (ADHD) [1,2,3,4,5]. Psychostimulants are the first choice medication for treating ADHD in children, adolescents, and adults [6]. Drug treatment has been used for over 50 years and leads to symptom reduction in about 70% of all patients [7,8]. In some cases, medication can lead to negative side effects, such as sleep disturbances, loss of appetite, stomach complaints, nausea, and vomiting. Many factors play a role in the pathophysiology and treatment of ADHD: genetics, neurology [9], and psychology [10], but also food intolerances [5,9,10,11,12,13,14,15,16,17,18,19,20,21,22,23,24,25,26,27,28,29,30,31,32]. Already 100 years ago, Shannon [10] observed increased restlessness and sleep disturbances in children with food intolerances. In 1961, the food intolerance hypothesis was supported by Crook et al. [33]. They determined that behavioral disorders can be caused by the ingestion of milk, cereals, eggs, or chocolate. It is known that children with ADHD differ in oligosaccharide metabolism [34], show more lactose intolerance [35], and have a higher incidence of celiac disease [36] as compared to healthy children. The role of the gut microbiome has been discussed [37,38]. In the early 1980s, Egger et al. proposed that food could trigger behavior problems [28]. Therefore, they used an oligoantigenic diet (OD) in children with the hyperkinetic syndrome. The OD is a diet that avoids certain foods that often trigger allergies or intolerances. Consequently, during the diet, participants eat foods that are proven to be hypoallergenic. The diet we used followed the food choices of Egger et al. [39] and Carter et al. [40], consisting typically of two types of meat (e.g., lamb and chicken), two carbohydrate sources (e.g., potatoes and rice), two fruits (e.g., banana and apple), vegetable (e.g., any brassica), calcium-rich water, and vitamin supplementation. The diet was adjusted to suit the tastes and habits of the family and to avoid any foods suspected of causing symptoms and those for which the child had a particular craving or dislike.

The Egger and Carter [28] study showed a symptom reduction in 62 (82%) participants and normal behavior in 22 (29%) participants at the end of the diet. An improvement in ADHD symptoms of about 40% in 60–80% of the participants has been shown in other previous studies, which seems to be due to intolerances to different food components [13,18,41].

In our open uncontrolled study, Yorgidis et al. could replicate and confirm previous results of the efficacy of the OD on children with ADHD in a pre-post design [42,43]. The participants showed individual food sensitivity concerning the type of food, intensity, or pattern of reactivity. In all patients, ADHD symptoms were intensified by various foods during the food challenge after OD. Because every participant reacted very individually to different foods, there must be an individual dietary recommendation for each individual child. Stevenson et al. [13] pointed out in their research review from 2014 that a restricted elimination diet might be beneficial for ADHD symptoms in children and adults. Subsequently, detected individual food sensitivities leading to individualized dietary recommendations are useful as an additional option to the existing multimodal therapy concept.

The purpose of our present follow-up analyses was to investigate if there is long-term success in an OD with subsequent individual dietary recommendations since it is important to achieve long-term treatment effects in ADHD [44]. For this purpose, follow-up data of the participants who completed the OD were collected again approximately 3.5 years later.

## 2. Materials and Methods

The Ethics Committee of the University of Freiburg (application number 111/14) approved the study in accordance with the World Medical Association’s Declaration of Helsinki.

### 2.1. Participants

The inclusion criterion of the study was the diagnosis of ADHD according to the criteria of the DSM-IV [45] and the ICD-10 [46], with signed informed consent. The ages of participants were between 7 and 14 years. Participants who met one or more of the following conditions on the list were excluded: concomitant disease, neurological or organic comorbidities which cannot be subjected to dietary intervention; lack of compliance either of the parents or children; lack of reading or writing skills; concurrent drug therapy of ADHD or participation in other studies at the same time; children following a special diet (e.g., vegetarian, vegan).

The children underwent a detailed family and self-history. A physical examination was conducted. Parameters of medical and pharmacological history were assessed and the concomitant medical status was ascertained. Furthermore, neurological and internist statuses and vital signs were obtained. A checklist for food allergies and food intolerances was used. After a detailed medical examination, the ADHD diagnosis was confirmed with the Kiddie-SADS-Present and Lifetime Version (K-SADS-PL) [47]. A total of 28 children between 7 and 14 years old participated in the study between 2015 and 2019. During the diet phase, 12 participants either dropped out (*n* = 2) of the study or were considered non-responders (*n* = 10). Reductions in ADHD symptoms of 40% or more were seen in 17 participants (responders). Data from 21 children were available at follow-up. Extended travel time to the Medical Center in Freiburg was the main reason for non-attendance. In Table 1, an overview of the participants’ characteristics is given.

### 2.2. Outcome Measures

The ADHD rating scale (ARS) total score was used as the primary outcome measure [48,49,50,51]. It represents a valid instrument to assess the behavior of children and adolescents [48]. A senior child and adolescent psychiatrist evaluated the questionnaire at all examination points.

With this scale, the frequency of ADHD symptoms can be determined. It consists of a total of 18 questions that refer to ADHD symptoms according to DSM-IV. The answers to the questions should be related to the events and behaviors of the last week before the survey. The 18 questions are divided into 2 subscales for inattention and hyperactivity/impulsivity. Thus, three scores were formed: the ARS total score and two scores for the two subscales for inattention and hyperactivity/impulsivity.

Three questionnaires were used as secondary outcomes. “Quality of life (ILC) for children, adolescents and parents” was completed by the children themselves to assess the quality of life subjective. The proxy assessment of the parents serves as an objective comparison. The “Diagnostic System for Mental Disorders in Childhood and Adolescence—II Other-Report—Attention Deficit/Hyperactivity Disorder (DISYPS-II FBB-ADHD)” asks about the subscales inattention, hyperactivity, and impulsivity. The difference to the ARS is that hyperactivity and impulsivity can be rated separately. Finally, the “Child Behavior Checklist 4-18 (CBCL/4-18)” inquires about competencies and clinically relevant challenging behavior [43].

**Figure 1:**
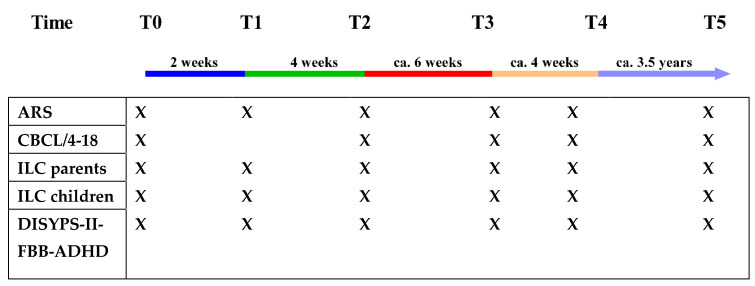
Timescales and measures for each appointment. Blue line: pre-diet phase; green line: diet phase OD; red line: reintroduction, testing the different main food groups, orange line: reconfirm different food intolerances, purple line: follow-up.

The primary and secondary outcomes were assessed at each of the time points mentioned in Figure 1 to obtain trajectories of the changes in the child’s symptoms and behavior.

### 2.3. Procedure

Figure 1 shows the individual phases of the study and the completed questionnaires at the respective time points.

#### 2.3.1. Baseline T0

Several cohorts entered the study between 2014 and 2017. At baseline, T0, a declaration of consent and a release of confidentiality between the physician and psychiatrist had to be available. The family- and self-history were taken at this time. Physical examinations were performed, during which blood was taken, pulse and blood pressure were measured, and weight and height were determined. In addition, specifics about health, food intolerances or allergies, and behavior were documented. The ADHD history, comorbidities, and infant development were acquired. By using the K-SADS-PL a psychiatric diagnosis for ADHD was confirmed by a psychiatrist.

#### 2.3.2. Pre-Diet Phase

After the first appointment (T0), a two-week phase followed in which the children were instructed to eat as usual (T0–T1), i.e., without any change in diet. Everything the child ate should have been recorded in detail by the parents in a food diary, including an exact description of the food and drinks, the form of preparation, and the list of ingredients for packaged foods, recipes, and spices.

#### 2.3.3. Diet Phase

During the four-week diet phase (T1–T2), the children were only allowed to consume oligoantigenic foods. The diet was implemented according to the protocols of the groups of Egger, Pelsser, and Buitelaar [4,28,29,30,31]. Oligoantigenic foods contain nutrients with low allergenic potential according to Egger and Pelsser [28,29]. A variety of foods should be used so far as they are practical to obtain and prepare. Parents and subjects were carefully instructed in the diet, a selection of suitable foods was presented, and a grocery list, as well as recipe suggestions, were handed out. Family members were advised to follow the diet with the child to increase adherence. Those who attended a full-time school were asked to eat home-prepared food. A professional nutritionist monitored the implementation of the diet to avoid any deficit of essential nutrients.

#### 2.3.4. Reintroduction Phase

In participants whose ARS value decreased by more than 40% between T1 and T2, the diet was classified as successful [27]. These participants began the reintroduction phase (T2–T3) to re-establish their usual diet by using the following scheme: Every three to four days, a new food item was reintroduced in the following order: milk products, favorite food, egg, grain products, fish, meat, vegetables, fruits, nuts, and so on. As soon as a change in behavior or other symptoms such as abdominal pain, headaches, or allergic reactions occurred after consumption, the child and adolescent psychiatrist was notified. The dietitian was informed of the symptoms that occurred and the triggering food item was to be avoided. Reactions to a food item occurred between ten minutes to several days after ingestion [28]. After reintroducing a new food item and checking what reactions it triggered, only tolerated and tested food items were to be consumed for 3 days. Depending on the number of foods tolerated or not tolerated, the duration of the reintroduction phase varied. The foods to which the participants reacted were tested again between T3 and T4. All other foods that were tolerated were included in the diet. After testing all foods, a final examination followed. Participants received an individual dietary recommendation and a list of foods to avoid. After following the recommendations for at least 1 year, intolerant foods could be reintroduced with close observation of the reactions.

#### 2.3.5. Follow-Up

The follow-up examination took place from 2.50 to 5.37 years after the start of the diet (*M* = 1422, *SD* = 346, *Mdn* = 1494, *IQR* = 536 days). Both responders and non-responders were re-interviewed in the follow-up study, as they benefited to a different extent from the diet. The acquired questionnaires are listed in Figure 1.

### 2.4. Statistical Analyses

Only participants with complete data sets were included in the statistical analyses. For statistical inference, a repeated-measures ANOVA (rmANOVA) was performed to test whether there was a difference between the different time points T1 (pre-diet), T2 (post-diet), and T5 (follow-up). Post hoc tests with Bonferroni correction were performed for comparisons between time points. In case of violation of the sphericity assumption, the degrees of freedom were adjusted using the Greenhouse–Geisser correction [52]. Effect size measures are reported in terms of estimated Cohen’s *d* according to the formula proposed by Morris and DeShon [53]. All statistical analyses were conducted with R (Version 4.1.1).

## 3. Results

### 3.1. Participants

Table 2 provides an overview of the participants who attended the follow-up study. Of 28 participants, 2 did not complete the diet and 21 (80.8% of the initial 26 participants who completed the diet) attended the follow-up. A total of 71.4% were male and 28.6% were female and the age ranged from 12 to 16. Of these 21 participants, 14 met the responder criterion and 7 did not. At follow-up, 28% of participants were taking medication. Table 3 shows how many responders/non-responders had medication intake.

### 3.2. ADHD Symptoms According to ARS

In total, the scores of 21 participants and, thus, 80.8% of the data of the entire dataset were available at follow-up. For these participants, the total scores and the scores of the two subscales inattention and hyperactivity/impulsivity were computed.

#### 3.2.1. ARS Total

**Figure 2:**
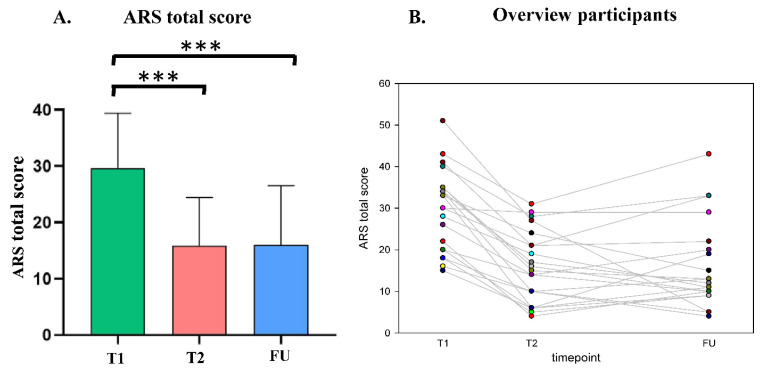
(**A**) Means and standard deviations of the ARS total score. T1 = start of the oligoantigenic diet, T2 = end of the oligoantigenic diet, FU = follow-up (*** *p* < 0.001) (**B**) ADHD rating scale for all the participants who attended the follow-up (*n* = 21), T1= start of the oligoantigenic diet, T2 = end of the oligoantigenic diet, FU = follow-up. (Each patient appears in different color).

For the ARS total score as shown in Figure 2 and Table 4, the results demonstrated, that the mean score improved from T1: *M* = 29.62 (*SD* = 9.80) to T2: *M* = 15.86 (*SD* = 8.56) between the time points before and after the diet. There was a lower ARS total score at follow-up T5: *M* = 16.00 (*SD* = 10.52) compared to the beginning of the diet. In the rmANOVA, a significant effect of time of measurement was obtained (*F*(1.36, 27.33) = 30.12, *p* < 0.001, Greenhouse–Geisser correction). Subsequent post hoc tests showed a significant improvement before the diet and follow-up (*p* < 0.001) and no significant improvement between the end of the diet and the follow-up (*p* > 0.999).

#### 3.2.2. ARS Inattention

The mean value of the subscale inattention at the beginning of the diet was T1: *M* = 16.52 (*SD* = 5.69) and decreased to T2: *M* = 8.29 (*SD* = 5.13) after the diet. At follow-up, the mean value was still lower than at the beginning of the diet, T5: *M* = 9.33 (*SD* = 5.00). The rmANOVA revealed a significant effect of time of measurement (*F*(2,40) = 23.51, *p* < 0.001). The post hoc test showed a significant improvement before the diet and the follow-up (*p* < 0.001) and no significant improvement between the end of the diet and the follow-up (*p* = 0.980).

#### 3.2.3. ARS Hyperactivity/Impulsivity

The mean score of the hyperactivity/impulsivity subscale decreased from before the diet, T1: *M* = 13.10 (*SD* = 6.00) to T2: *M* = 7.57 (*SD* = 5.65) after the diet. At follow-up, it was at T5: *M* = 6.67 (*SD* = 6.54). The rmANOVA revealed a significant effect of time of measurement (*F*(1.55, 30.92) = 21.22, *p* < 0.001, Greenhouse–Geisser correction) The post hoc test showed a significant improvement (*p* < 0.001) of the subscale between the time before the diet and the follow-up and no significant improvement between the diet and the follow-up (*p* > 0.999).

#### 3.2.4. Responders/Non-Responders

**Figure 3:**
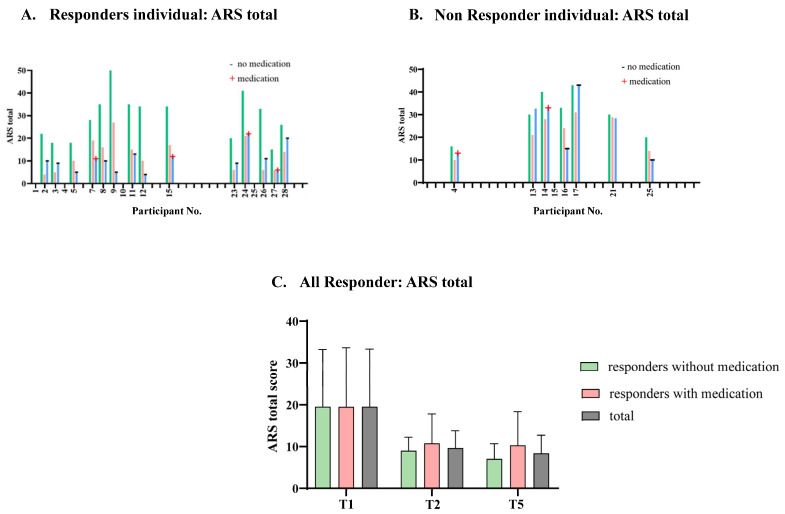
(**A**) Course of the ARS scores over time from the responders. Green = before the diet, red = after the diet, blue = follow–up. (**B**) Course of the ARS scores over time from the non-responders. Green = before the diet, red = after the diet, blue = follow–up; (**C**) means and standard deviations of all responders.

Responders were participants in the study whose ARS total scale scores decreased by 40% between the time before the diet (T1) and the end of the diet (T2). Of the 21 participants at follow-up, 14 were responders, representing a responder rate of 66.67% (see Table 1). Seven participants were non-responders (33.33%). After an average of 3.5 years, all responders showed symptom reduction in comparison to before the diet. Four of the responders had medication intake. Of the 14 responders at follow-up with individual dietary recommendations, 4 were still on the diet and without medication (ARS total: 5, 13, 4, 11). A total of 10 responders reported an attempt to reintroduce intolerant foods. Of these, 5 reported problems after intake, 3 reported stomach pain, 1 experienced exhaustion after milk, 1 had attention problems after corn. Those with medication (*n* = 4) did not keep the diet due to either the number of intolerances, exclusion by peers, or a successful drug trial. The results indicate that at least 10 of 28 children, more than 30% of the children included in the beginning, benefit from OD intervention long-term. The mean ARS total score for the responders decreased over the time points. If we exclude the responders with medication from our analysis, the ARS total scores are even better. In Figure 3, the means are compared. For the non-responders, the following means over the three time points also show an improvement in ARS total score (T1: *M* = 30.28, *SD* = 9.05; T2: *M* = 22.42, *SD* = 7.34; T5: *M* = 25.14, *SD* = 11.56). Although there was an increase in the score between the time points after the diet and the follow-up, the score at follow-up was lower than before the diet. Figure 3A,B show the course of ARS total score over time. Figure 3C breaks down the ARS total score for the total sample of responders and those with or without medication at times of measurement before the diet, after the diet, and at follow-up.

### 3.3. Secondary Outcomes

The secondary outcome parameters dertermined in the present study are presented in Table 5. For the CBCL/4-18, which assesses behavioral problems, emotional problems, somatic complaints as well as social skills, data from 19 participants were available at follow-up. Results from rmANOVA showed that significant improvements could be obtained in some scales only. Significant improvements between (T1) and (T5) were obtained for the following subscales: total (T1/T5: *p* = 0.001), external (T1/T5: *p* < 0.001) and attention problems (T1/T5: *p* = 0.003). In the following scales, there were no significant improvements after the diet and at follow-up: total (T2/T5: *p* = 0.890), external (T2/T5: *p* = 0.150), and attention problems (T2/T5: *p* > 0.999). The internal, social retreat, physical complaints, social problems, and schizoid obsessions of the scales did not show significant improvements. Children’s quality of life was rated by parents on the one hand and by the children themselves on the other. Data from 18 participants were available for the evaluation of the parents’ ILC. For the children’s self-report, data from 16 participants were available. In general, the parents gave better ratings than the children. The parents’ proxy rating in quality of life showed significant improvement in the item ‘friends’ (T1/T5: *p* = 0.010) and ‘body’ (T1/T5: *p* = 0.040) between the measuring times before the diet and the follow-up. There were no significant improvements in these two items after the diet and at follow-up: ‘friends’ (T2/T5: *p* > 0.999), ‘body’ (T2/T5: *p* = 0.170). In contrast, in the children’s perspective, only the item ‘friends’ improved significantly between the time before the diet and the follow-up (T1/T5: *p* = 0.022), but not significantly between the time after the diet and the follow-up (T2/T5: *p* > 0.999). Parents’ ratings in the DISYPS-II-FBB-ADHD confirmed the results of the ARS parent questionnaire. Both the total score (T1/T5: *p* < 0.001) and the scales inattention (T1/T5: *p* < 0.001), hyperactivity (T1/T5: *p* < 0.001), and impulsivity (T1/T5: *p* < 0.001) showed significant improvements in the time between the time before the diet and the follow-up. However, they did not show a significant improvement between the time after the diet and the follow-up: total score (T2/T5: *p* > 0.999), inattention (T2/T5: *p* > 0.999), hyperactivity (T1/T5: *p* > 0.999), impulsivity (T2/T5: *p* = 0.300). An overview of the results of the secondary outcomes is given in Table 5.

## 4. Discussion

The main goal of our study was to investigate whether the OD leads to an improvement of ADHD symptoms in children with ADHD after an average of 3.5 years. The results suggest that the oligoantigenic diet has a long-term positive effect on ADHD symptoms at least in some children. Since this is the first study on the long-term outcomes after oligoantigenic diets in children with ADHD, there are no data to compare with. At least 10 children did not switch to medication, this is about one-third of the initial study population.

In this study, we demonstrate again a significant reduction of ADHD symptoms directly after a four-week OD treatment. As in our primary study [43], we were able to replicate the positive effects of such dietary interventions described in previous studies [14,28,31,54,55,56,57].

Though the study was not blinded, the observed responder rate of about 64% after four weeks of diet was similar to the results of Egger [28] and Pelsser [29], who also observed responder rates of about 60%; Boris and Mandel [41] reported a 73% response rate. In the study by Schmidt et al. [58], a responder rate of 24% was reported, but a very short diet of only 9 days was used. Moreover, differences in the study design might contribute to the variation in responder rates. In this study, the response was defined as a 40% decrease in ARS total value, referring to Pelsser [31]. For all of those, intolerant food items could be identified. In our previous study, significant improvement was observed in all scales of the ADHD rating scale as the primary outcome measure [42].

The CBCL/4-18, the leading screening questionnaire to score a child’s mental health [59,60,61], gave a broad overview of the various psychological symptoms of the participants. Behavioral and emotional problems of the child were obtained as well. Parents reported a significant recovery in children’s mental and physical health as well as in social interactions after four weeks of OD. The strongest effects could be noted in attention problems at the end of the diet. These effects were also present at follow-up. After approximately 3.5 years, parents still reported significant improvements in children’s attention problems. Concerning the ILC questionnaire, as a measure of the quality of life, self-ratings showed the highest effects after the diet for the items ‘other children’ and ‘alone’. Schwörer et al. [62] reported the strongest effects of drug treatment on the item ‘school’. The answers of the parents showed similar effects on all items as reported for methylphenidate treatment by Berek et al. [63]. The DISYPS-II-FBB-ADHD [64], a valid tool to assess the effects of intervention on ADHD symptoms, allows the scoring of hyperactivity and impulsivity separately [65]. The results of this questionnaire, filled out by the parents only, support our results with the primary outcome ARS. Significant reductions were present in all subscales of the DISYPS-II-FBB-ADHD when subjects were abstaining from non-tolerated foods. Effects are similar to the use of multimodal therapy as reported by Meßler et al. and the implementation of neurofeedback as demonstrated by Gevensleben et al. [66,67]. We were able to show significant results not only between the time before and after the diet but also between the time before the diet and the follow-up examination. These outcomes support our results from the primary outcome ARS. The underlying mechanisms for the observed effects on ADHD symptoms remain elusive. One might speculate about immunological [68,69], vagal [70,71], or even microbiological influences [37,38,72,73,74,75,76,77,78] contributing to the interaction of gastrointestinal tract and brain. Nevertheless, it might also be possible that special food components directly interact from the mouth to the brain. Allergic reactions to food, clearly correlated to immunological mechanisms, often occur at first contact in the mouth (for review see [79]). All 10 responders without medication at follow-up had an ARS total value of about 10, with one exception (Participant 28). In the ILC Children, we see that the items ‘alone’ and ‘other children’ were still the items with major improvements. The parents report in their ILC further improvements at follow-up for the items ’alone’, ‘school’, ‘family’, and ‘other children’. Using the CBCL/4-18, they reported further improvements in the total score especially for the items ‘social problems’, ‘external’, and ‘attention’. This may demonstrate that at least 10 of 28 children, more than 30% of the children included in the beginning, benefit from OD intervention long-term.

## 5. Limitations and Future Directions

The study was open and non-randomized, there was no control group, families self-selected the oligoantigenic diet, and the diet was conducted without blinding. The number of participants in the study was relatively small. Although we replicated the effects from previous studies [13,41,42,43], a larger group of participants is needed to corroborate the results in future studies.

The assessment of daily behavior could be influenced and biased by various factors such as personal mood, physical health, or social interactions. Moreover, the severe changes in daily life and the explicit focus thereby on behavior may lead to bias in the assessment. Pandemic-related school closings with the need for homeschooling occurred during the follow-up. This might have influenced the assessment.

In the future, it should be investigated by which mechanism the different foods trigger an intensification of ADHD symptoms. The results of children taking medications in parallel with the diet can be very promising too.

## 6. Conclusions

ADHD symptoms and other clinical abnormalities can be improved by an oligoantigenic diet. It can be a treatment option for ADHD not only in the short term but also in the long term. Food intolerances are individual and the oligoantigenic diet is currently the gold standard to identify them. Personalized nutrition could be a valid tool for the personalized treatment of ADHD.

## Figures and Tables

**Table 1:** Characteristics of participants at (1) end of the OD intervention and (2) at follow-up approximately 3.5 years later.

	End of OD Intervention	Follow-Up
Included (*n*)	28	21
Age (*M ± SD* (range))	9.25 ± 1.73 (7–13)	13.94 ± 2.14 (12–16)
Gender (m/f)	21/7	15/6
Comorbidities (Diagnosis code ICD-10, *n*)	- Dyslexia (F81.0, *n* = 7) - Dyscalculia (F81.2, *n* = 2) - Oppositional defiant disorder (F91.3, F91.8, *n* = 2) - Autism (F84.0, *n* = 2) diagnosed in the course of the study - Encopresis (F98.1, *n* = 1) - Psoriasis (L40, *n* = 1) - Somnambulism (F51.3, *n* = 1) - Depersonalization and derealization syndrome (F48.1, *n* = 1) - Asperger’s syndrome (F84.5, *n* = 1)	- Dyslexia (F81.0, *n* = 5) - Oppositional defiant disorder (F90, *n* = 1) - Enuresis (F98, *n* = 2) - Expressive language disorder (F80.1, *n* = 1) - Adjustment disorder (F43.2, *n* = 1) - Psoriasis (L40, *n* = 1) - Somnambulism (F51.3, *n* = 1) - Depersonalization and derealization syndrome (F48.1, *n* = 1) - Asperger’s syndrome (F84.5, *n* = 1)

**Table 2:** Frequency distribution of children with ADHD that followed an oligoantigenic diet (FU = follow-up, 2–5 years after start).

Participants	Diet (*n*)	Percentage
Starting diet	28	100%
Completed diet	26 (28)	92.8%
Drop-out	2 (28)	7.14%
	**FU (*n* (*end of diet*))**	**Percentage**
Feedback at follow-up	21 (26)	80.8
Responders	14 (17)	66.7 (65.4)
Non-Responders	7 (9)	33.3 (34.6)

**Table 3:** Medication of the responders and non-responders at follow-up (FU).

Medication	Responders (*n*)	Percentage (%)	Non-Responders (*n*)	Percentage (%)
Yes	4	28.6%	2	28.6%
No	10	71.4%	3	42.8%
No Answer	0	0%	2	28.6%

**Table 4:** Means, standard deviations, repeated-measures analyses of variance, and post hoc comparisons with effect sizes for the scales of the primary outcome measure ADHD Rating Scale (*n* = 21).

	Pre		Post		FU		*F*	*p*	Pre vs. Post		Pre vs. FU		Post vs. FU	
	*M*	*SD*	*M*	*SD*	*M*	*SD*			*r*	*d*	*r*	*d*	*r*	*d*
ARS-Total	29.62	9.80	15.86	8.56	16.00	10.52	30.12	<0.001	0.730	−1.91 ***	0.298	−1.17 ***	0.637	0.02
ARS Inattention	16.52	5.69	8.29	5.13	9.33	5.00	23.51	<0.001	0.456	−1.39 ***	0.083	−0.93 ***	0.551	0.21
ARS H/I	13.10	6.00	7.57	5.65	6.67	6.54	21.22	<0.001	0.825	−1.56 ***	0.567	−1.15 ***	0.663	−0.19

*Note*. Pre = pre-diet T1; Post = post-diet T2; FU = follow-up T5; *r* = Pearson correlation between repeated measurements; *d* = Cohen’s *d* estimated by the formula of Morris and DeShon (2002). ARS-Total = ADHD-Rating-Scale-IV—Total; ARS-Inattention = ADHD-Rating-Scale-IV—subscale inattention; ARS H/I = ADHD-Rating-Scale-IV—subscale hyperactivity/impulsivity. *** *p* < 0.001.

**Table 5:** Means, standard deviations, repeated-measures analyses of variance, and post hoc comparisons with effect sizes for secondary outcome measures.

		Pre		Post		FU		*F*	*p*	Pre vs. Post		Pre vs. FU		Post vs. FU	
	*n*	*M*	*SD*	*M*	*SD*	*M*	*SD*			*r*	*d*	*r*	*d*	*r*	*d*
CBCL/4-18	19	66.79	5.95	60.21	6.38	58.21	7.73	14.56	<0.001	0.749	−1.56 ***	0.254	−1.18 **	0.343	−0.27
ILC Parents	18	17.67	2.81	20.39	3.53	18.83	3.24	6.81	0.003	0.589	1.07 **	0.453	0.40	0.535	−0.46
ILC Child	16	20.31	3.61	20.81	3.92	21.19	3.12	<1	0.699	0.626	0.16	0.101	0.18	0.215	0.07
DISYPS-II-FBB-ADHD	17	1.61	0.50	0.80	0.45	0.72	0.34	40.77	<0.001	0.722	−2.17 ***	0.251	−1.45 ***	0.383	−0.16

*Note*. Pre = pre-diet T1; Post = post-diet T2; FU = follow-up T5; *r* = Pearson correlation between repeated measurements; *d* = Cohen’s *d* estimated by the formula of Morris and DeShon, 2002. DISYPS-II-FBB-ADHD = total score of the diagnostic system for mental disorders in childhood and adolescence—II Other-Report—attention-deficit/hyperactivity disorder; CBCL/4-18 = child behavior checklist 4-18—total score; ILC Parents = inventory of life quality in children and adolescents—total score of parents’ proxy report; ILC Child = inventory of life quality in children and adolescents—total score of child’s self-report. ** *p* < 0.01. *** *p* < 0.001.

## Data Availability

The data are not publicly available according to the description of confidentiality and data sharing procedures described in the study’s informed consent and assent documents.
